# Supplementary material for: Whole-genome sequence analysis unveils different origins of European and Asiatic mouflon and domestication-related genes in sheep
Source: Commun Biol. 2021 Nov 18;4:1307. doi: 10.1038/s42003-021-02817-4 (PMC8602413; doi:10.1038/s42003-021-02817-4)
Supplement: Supplementary file 1 — Supplementary information [file 42003_2021_2817_MOESM1_ESM.pdf]

Supplementary Information for

Whole-genome sequence analysis unveils different origins of European and Asiatic mouflon and domestication-related genes in sheep

Ze-Hui Chen, Ya-Xi Xu, Xing-Long Xie, Dong-Feng Wang, Diana Aguilar-Gómez, Guang-Jian Liu, Xin Li, Ali Esmailizadeh, Vahideh Rezaei, Juha Kantanen, Innokentyi Ammosov, Maryam Nosrati, Kathiravan Periasamy, David W. Coltman, Johannes A. Lenstra, Rasmus Nielsen*, Meng-Hua Li*

*To whom correspondence may be addressed. Email: menghua.li@cau.edu.cn (M.H. L.) or rasmus_nielsen@berkeley.edu (R.N.)

This PDF file includes:

Supplementary Results

Supplementary Figs. 1–18

Supplementary Tables 1–7

Supplementary References

Supplementary Results

Abundance and Annotation of structural variations (SVs)

The average abundance of SVs per individual ranged from 37,108 (±1,639) in urial to 46,946 (±4,323) in Asiatic mouflon (Supplementary Data 2). We observed 20,976 SVs shared among all the species, with the highest number of species-unique SVs in Asiatic mouflon (n = 47,184) and the lowest in European mouflon (n = 3,131) (Supplementary Data 2). Similarly, the number of species-unique CNVs varied from 776 in European mouflon to 18,032 in Asiatic mouflon, and a total of 16,016 CNVs were found to be shared among all the species (Supplementary Data 2). The average number of CNVs per individual ranged from 27,608 (±679) in European mouflon to 34,448 (±1,598) in thinhorn sheep (Supplementary Data 2). Moreover, there were 61,125 (23.3%) species-unbalanced SVs that were distributed unevenly among the species (Supplementary Fig. 3b).

We found 3,023 functional genes overlapping with shared SVs regions among all eight species. The top 15 significant Gene Ontology (GO) terms and Kyoto Encyclopedia of Genes and Genomes (KEGG) pathways for these genes were enriched for neural signal transmission, circadian entrainment, oxytocin and calcium signaling pathways. GO and KEGG pathway analyses of the 3,279 genes in the unique SVs regions identified in at least two of the 16 Asiatic mouflons were significantly ($P < 0.01$) enriched for vascular smooth muscle contraction, neuro signal transduction, and calcium and ATP binding. In domestic sheep, we observed a total of

2,068 genes in the unique SVs region, and the genes were significantly ($P < 0.01$) enriched for signal transduction, cardiomyopathy, cell and focal adhesion. Of the 999 genes in snow sheep, the genes were significantly ($P < 0.01$) enriched for long-term potentiation, which relates to long-term memory (e.g., *GRM5*), long-term depression (e.g., *PLCB1*), renin secretion and circadian entrainment (e.g., *PRKG1* and *RYR2*) [1-5]. Of the 708 genes in thinhorn sheep, genes were significantly ($P < 0.01$) enriched for startle response, calcium and ion channel activities, and bacterial invasion (Supplementary Data 2).

linkage disequilibrium (LD) of sheep species

LD decayed to its half maximum within <10 kb for wild species, whereas it decayed at ~10 – 90 kb for domestic sheep. This LD decay pattern could be explained by a stronger bottleneck effect in sheep during domestication than in the wild species. For domestic populations, LD differed significantly with the half maximum ranging from ~10 kb in Mazekh sheep to ~90 kb in Ouessant sheep (Fig. 3b).

Supplementary Figures

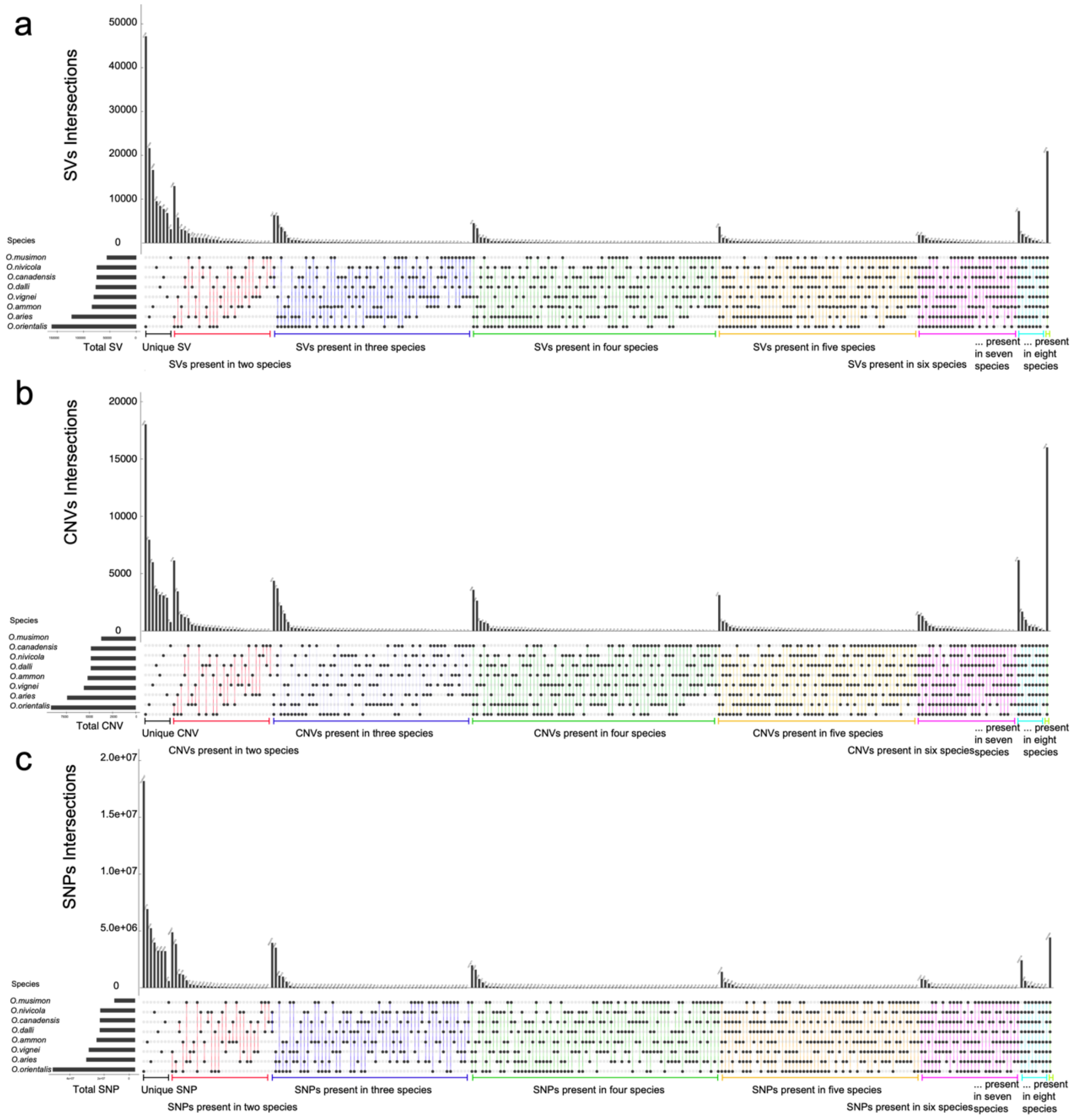

Supplementary Fig. 1 Upset plot of SVs, CNVs and SNPs. **(a)** Per set intersection of SVs. **(b)** Per set intersection of CNVs. **(c)** Per set intersection of SNPs. Different colors mean uniqueness and sharing among eight combination sets.

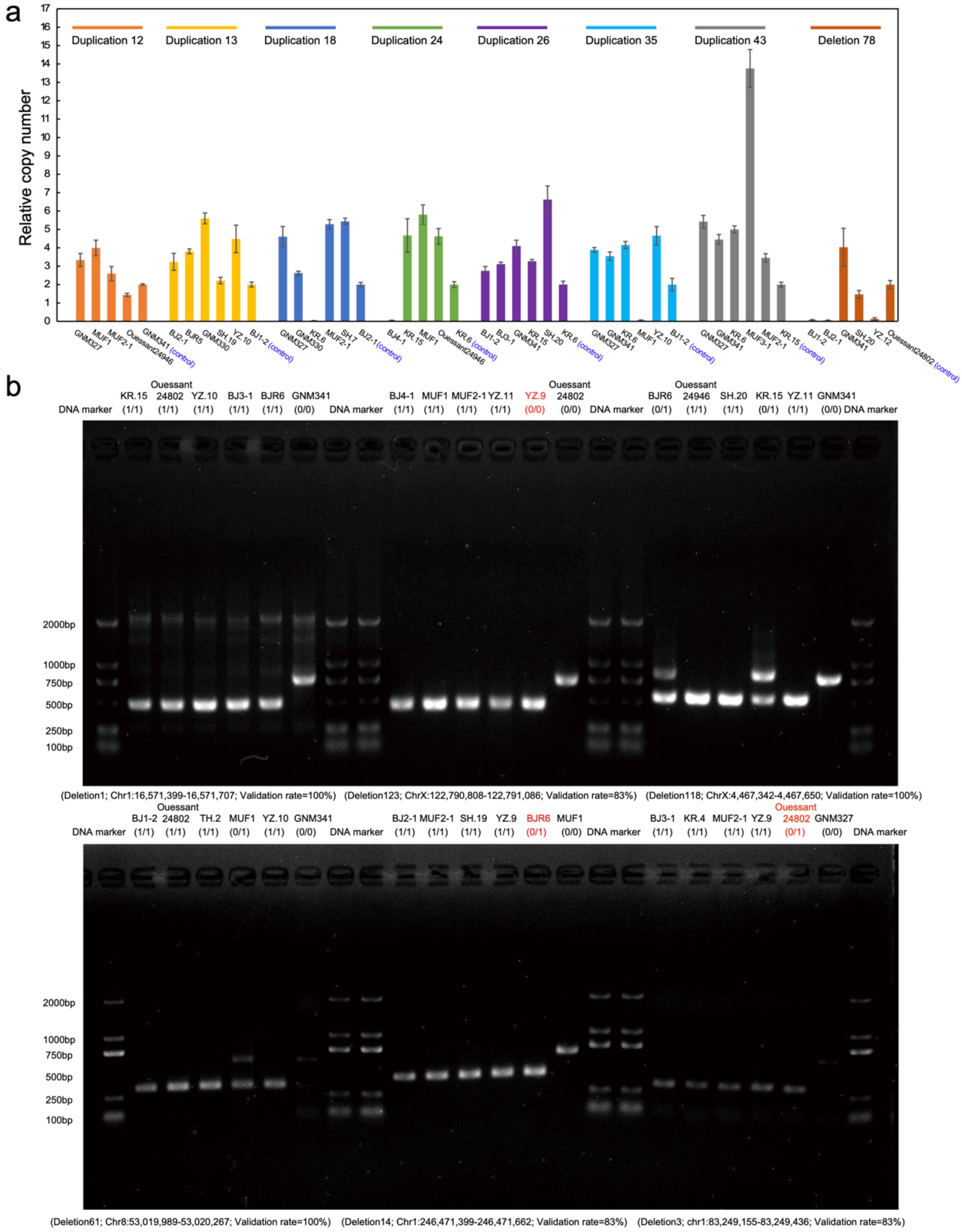

Supplementary Fig. 2 CNVs validation by qPCR and PCR. **(a)** Relative quantification of six duplications and one deletion by qPCR. The x and y axis represent sample ID and relative CNVs expression level (mean±SE, n=3) **(b)** Six genotyped deletions validated by regular PCR and checked through the length of the PCR products by gel assays. Source data are provided in Supplementary Data 3.

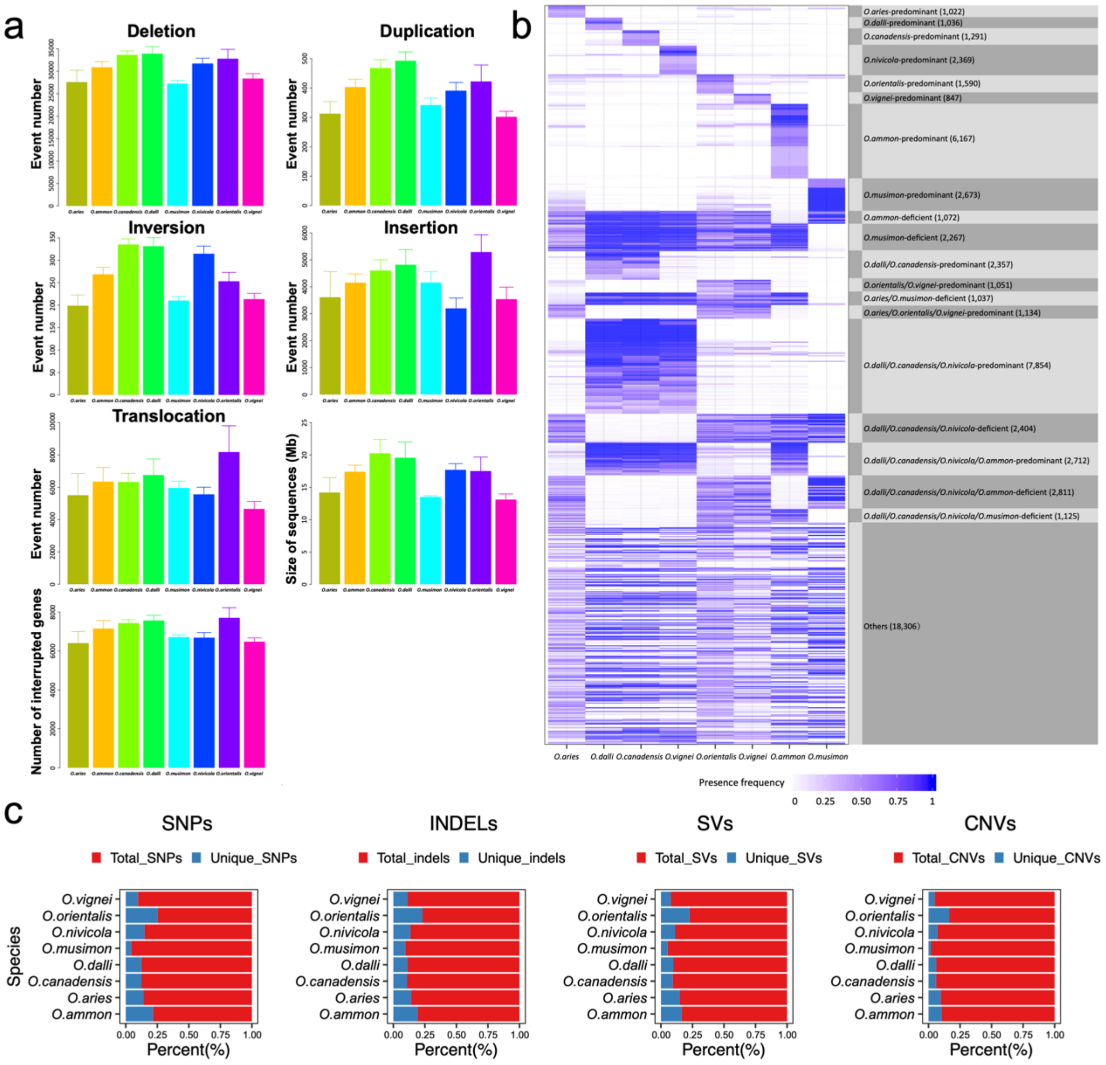

Supplementary Fig. 3 Statistics of variations (SNPs, Indels, SVs, CNVs) of *Ovis* species. **(a)** Statistics of structural variation (deletion, duplication, inversion, insertion and translocation) of each species in *Ovis* genus. Size of sequence is the genome sizes affected by SVs. Histogram of the fourth row is the numbers of genes affected (included or interrupted) by the SVs. Bar plots are mean ± s.d. **(b)** Characterization of the major-group-unbalanced SVs unevenly distributed among all the species on the basis of two-sided Fisher's exact tests. **(c)** Total and unique counts of SNPs, INDELs, SVs and CNVs in all species of *Ovis*. Here, eight species were counted: urial (*O.vignei*), Asiatic mouflon (*O.orientalis*), European mouflon (*O.musimon*), argali(*O.ammon*), domestic

sheep (*O.aries*), snow sheep (*O.nivicola*), bighorn (*O.canadensis*), and thinhorn (*O.dalli*).

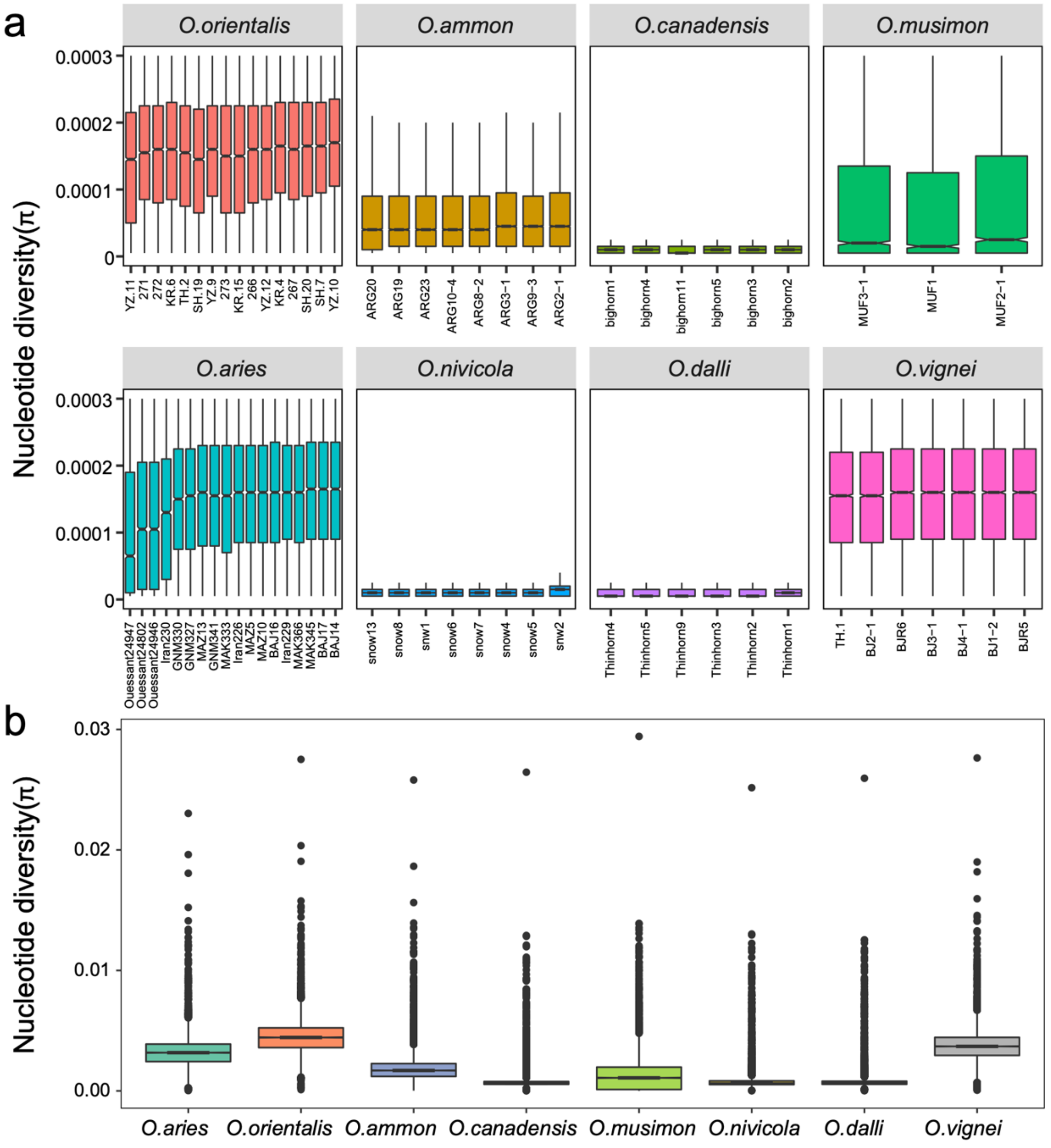

Supplementary Fig. 4 Nucleotide diversity of *Ovis* species based on sets of SNPs with 200-kb sliding window size. **(a)** Nucleotide diversity of individuals in groups of eight species [urial (*O.vignei*), Asiatic mouflon (*O.orientalis*), European mouflon (*O.musimon*), argali(*O.ammon*), domestic sheep (*O.aries*), snow sheep (*O.nivicola*), bighorn (*O.canadensis*), and thinhorn (*O.dalli*)]. **(b)** Average nucleotide diversity of each species. The top, middle (within the box), and bottom boundary lines of the boxes represent 25%, 50% (median value), and 75% of π.

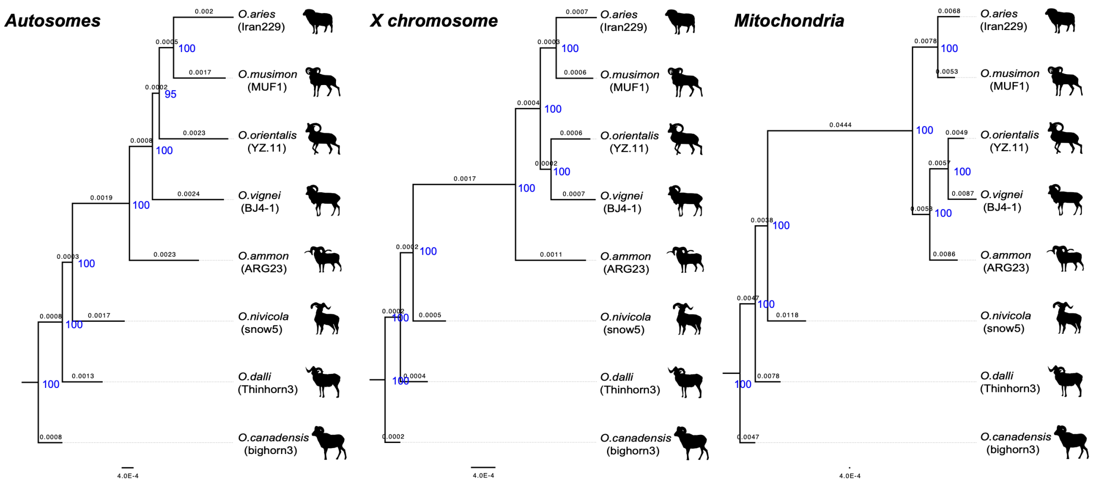

Supplementary Fig. 5 Phylogenetic trees of autosomes, X chromosome and mitochondria using haploidized genomes. One high-depth individual of each species were selected for analysis. Only concatenated protein coding sequences (CDS) are used for tree construction. Branch length were marked upon branches with numbers, the scale bar indicates the scale of branch length. Bootstrap values were marked in blue. Here, we take bighorn as the root group.

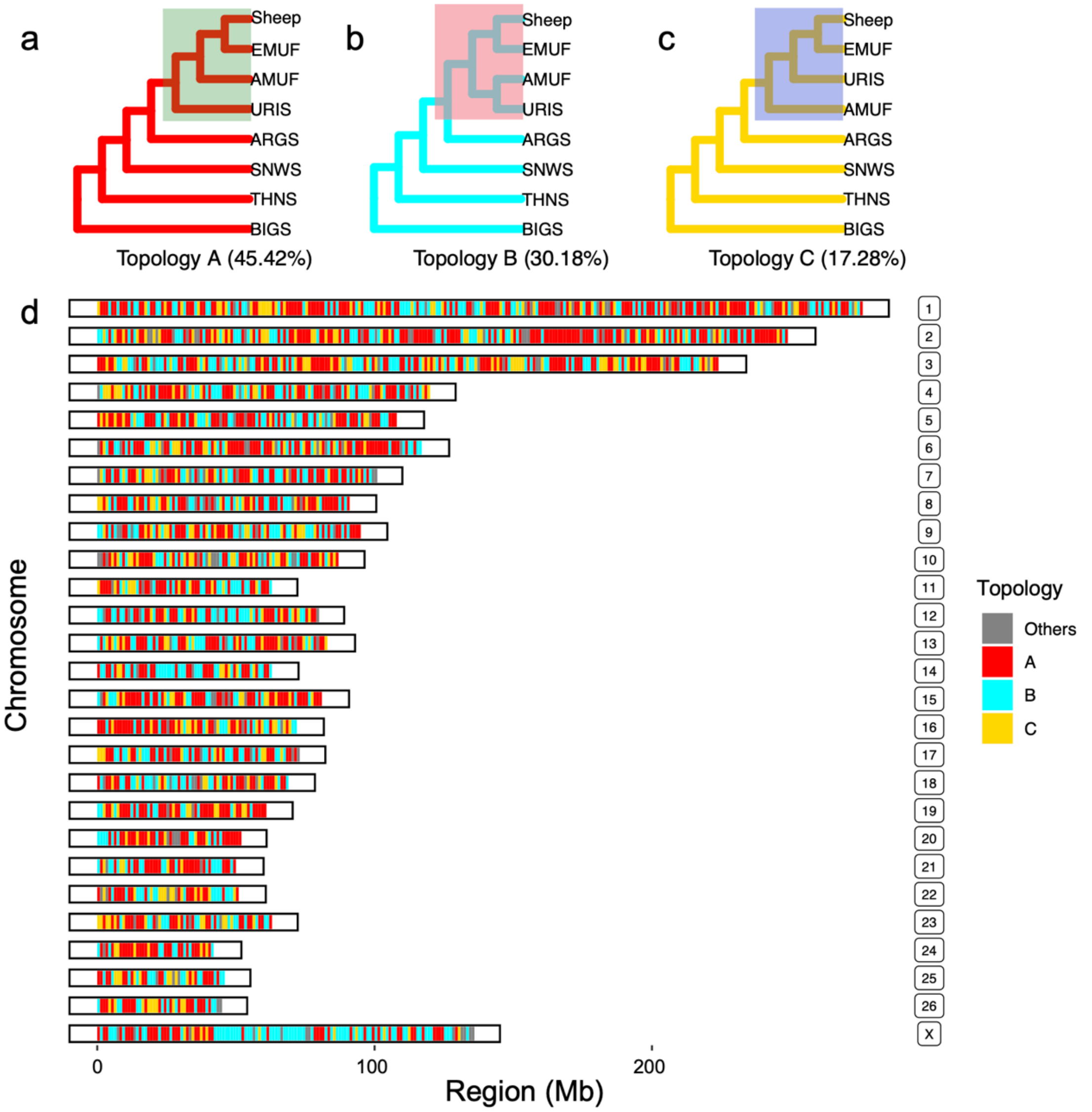

Supplementary Fig. 6 Chromosome painter of top 3 topologies across all 1-Mb non-overlapping sliding windowed trees. **(a)** The top one **(b)** top two and **(c)** top three tree topology in the whole genome. The colors of the chromosome segments are corresponding to the top three domain tree topologies. Shadows marked on each topology imply an inconstant phylogeny.

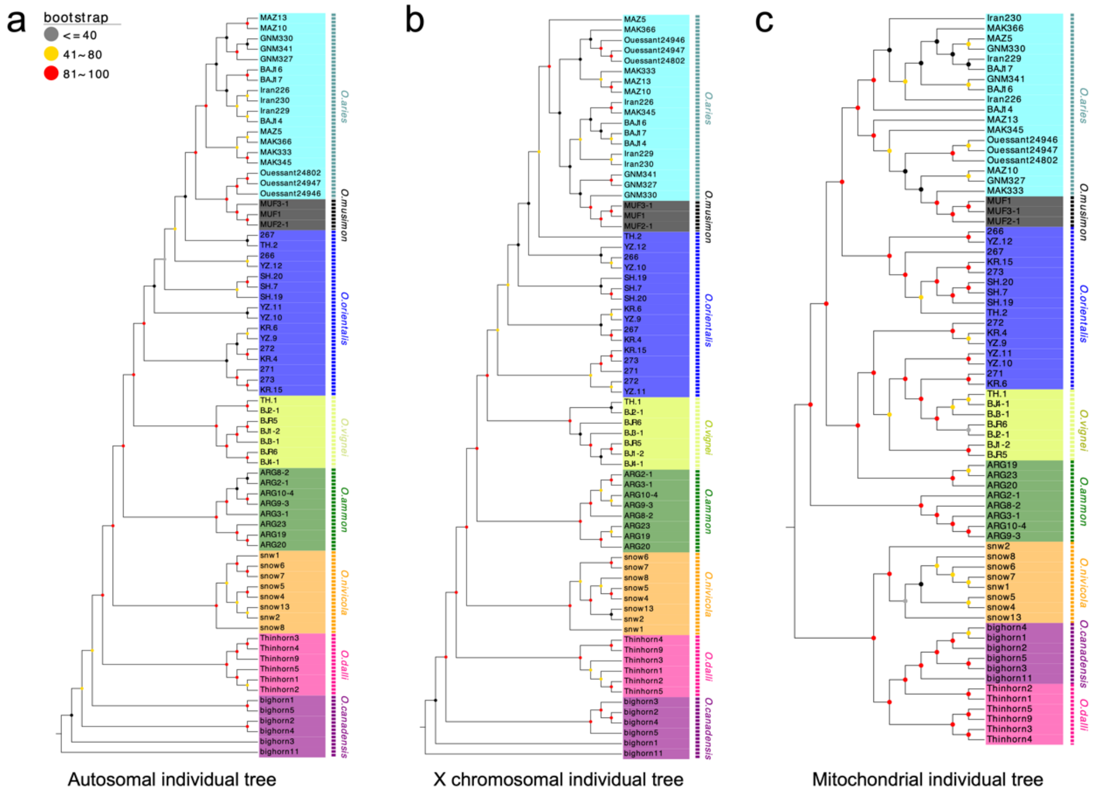

Supplementary Fig. 7 Individual trees of autosomes, X chromosome and mitochondria. **(a)** Autosomal tree without root, each color indicates one species. **(b)** X chromosomal tree without root. **(c)** Mitochondrial tree was rooted with the goat outgroup which then was trimmed. Here the full mitochondrial sequences were aligned with the goat outgroup, while only the protein coding sequences were aligned for 72 individuals on autosomes and X chromosome. Range of bootstrap value were marked on each node.

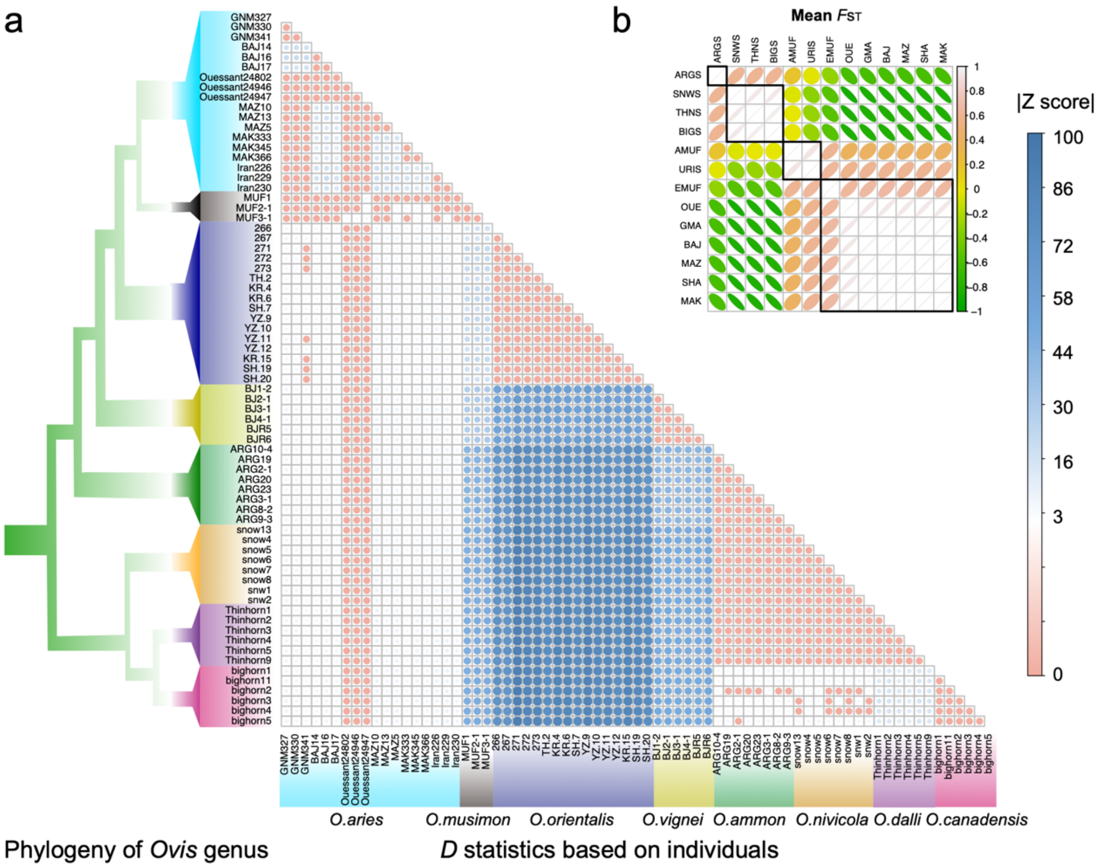

Supplementary Fig. 8 Genetic correlation with the *Ovis* genus. **(a)** Matrix plot for |Z score| of *D* statistics on individual bases using whole genome SNPs dataset, Z scores of each pair used in the plot are shown in Supplementary Data 4. |Z score| >3 or <3 are plotted as blue or red dots, respectively. The phylogeny of *Ovis* species on autosomes was plotted in the left of the matrix plotting. **(b)** Correlations based on the pairwise F_{ST} estimation.

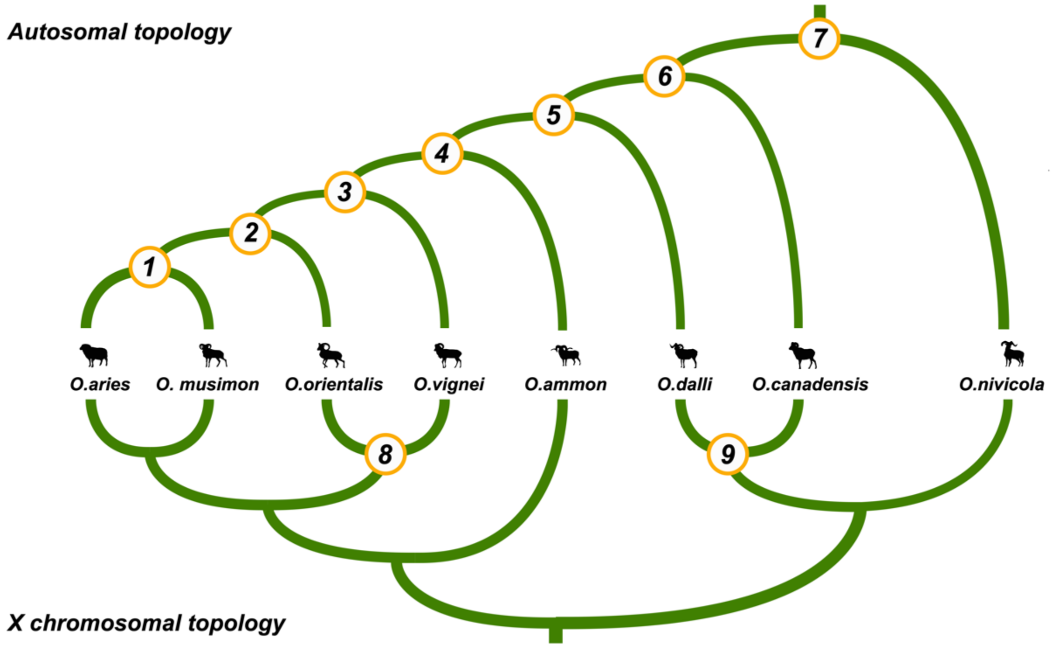

Methods	1	2	3	4	5	6	7	8	9
CoalHMM (I model)	5554 [5213,5896]	8843 [-14234,31921]	92520 [89807,95233]	150039 [148530,151549]	244488 [242320,246656]	246081 [243894,248268]	243616 [241412,245820]	83134 [80460,85808]	35848 [34510,37187]
CoalHMM (IM model)	5450 [5197,5703]	12838 [11681,13994]	77163 [72305,82021]	124268 [117955,130581]	265284 [255160,275408]	270081 [260124,280037]	261305 [251684,270925]	73017 [68885, 77150]	51656 [42686,60626]

Supplementary Fig. 9 Tree topologies on autosomes and X chromosome and divergence time (95 % confidence interval) inferred by CoalHMM program. I model: Compete isolation model; IM model: Isolation with migration model.

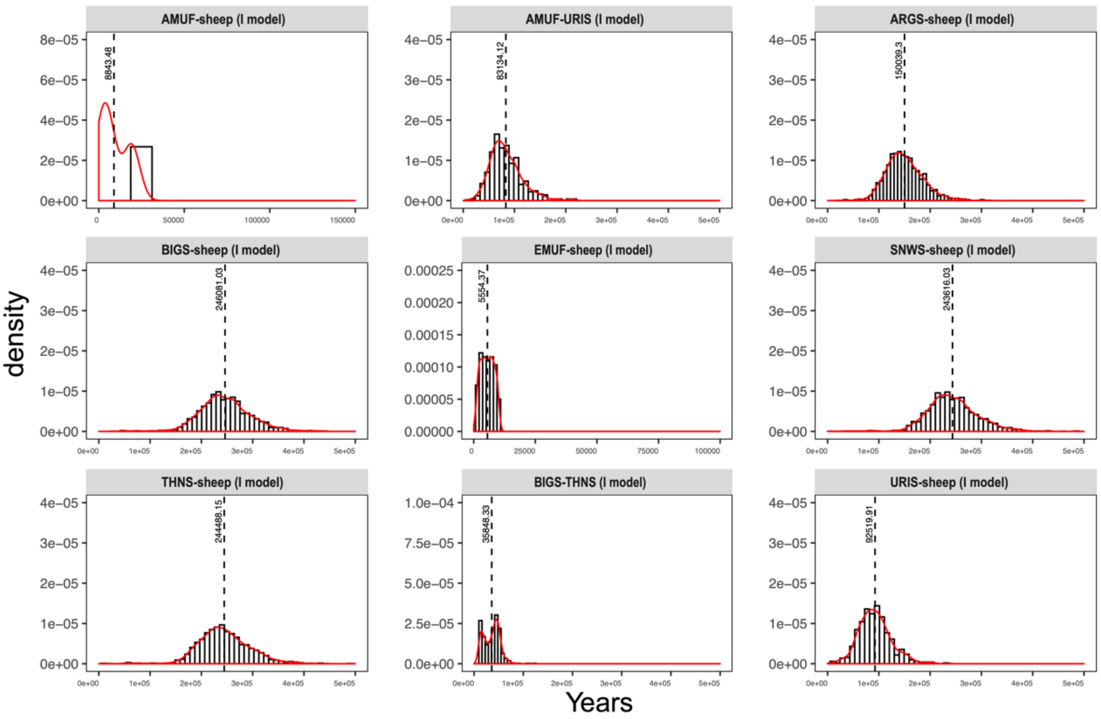

Supplementary Fig. 10 Density plot of divergence time from 9 pairwise comparison estimated by complete isolation model (I model) in CoalHMM program. Vertical dotted lines were the average value of distribution in each pair. Genomes in each pair were split into 1-Mb windows. Divergence time were calculated windows by windows and time of windows below 10,000 years or above 10 million years were filtered. Particularly, time of windows below 1,000 or above 10,000 for the pair of European mouflon and sheep, below 1,000 or above 20,000 for the pair of Asiatic mouflon and sheep were removed.

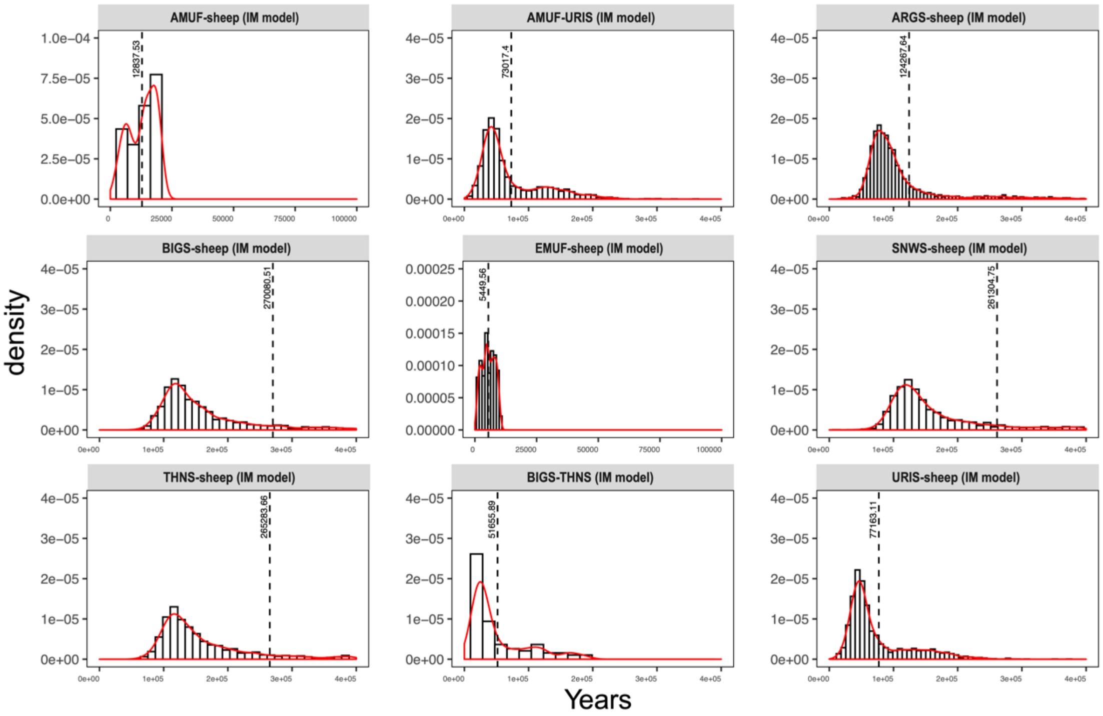

Supplementary Fig. 11 Density plot of divergence time from 9 pairwise comparison estimated by isolation with migration model (IM model) in CoalHMM program. Vertical dotted lines were the average value of distribution in each pair. Genomes in each pair were split into 1-Mb windows. Divergence time were calculated windows by windows and time of windows below 10,000 years or above 10 million years were filtered. Particularly, time of windows below 1,000 or above 10,000 for the pair of European mouflon and sheep, below 1,000 or above 20,000 for the pair of Asiatic mouflon and sheep were removed.

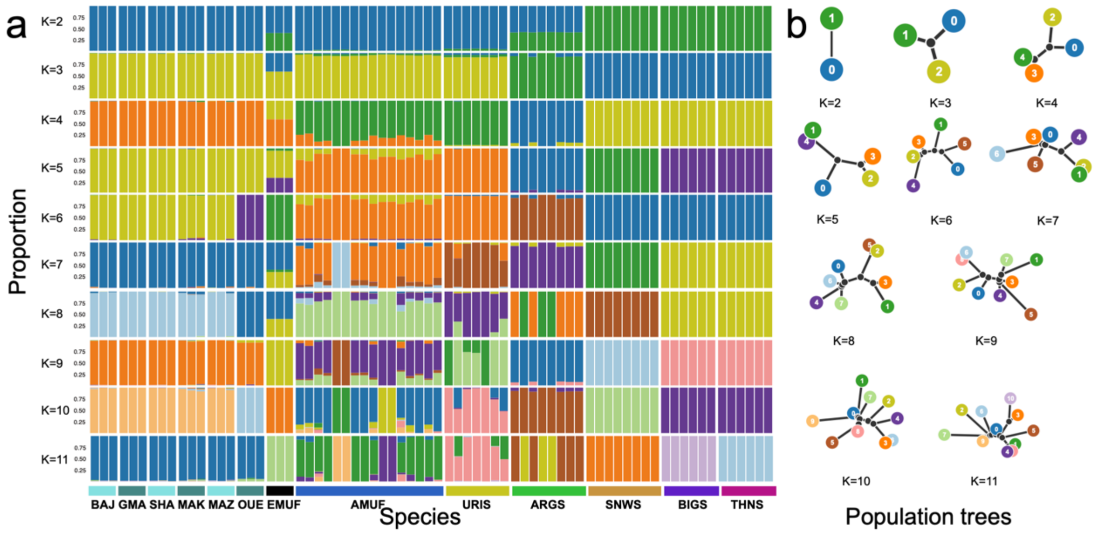

Supplementary Fig. 12 Admixture plot **(a)** while taking K from 2 to 11. **(b)** The population trees of each K. Tree topologies of each K indicate affinity of each ancestral component. Here, BAJ is Baidarak sheep (*O.aries*), GMA is Tibetan sheep (*O.aries*), SHA is Shal sheep (*O.aries*), MAZ is Mazekh sheep (*O.aries*), MAK is Makui sheep (*O.aries*), OUE is Ouessant sheep (*O.aries*), EMUF is European mouflon (*O.musimon*), AMUF is Asiatic mouflon (*O.orientalis*) , URIS is urial (*O.vignei*), ARGS is argali (*O.ammon*), SNWS is snow sheep (*O.nivicola*), BIGS is bighorn (*O.canadensis*), and THNS is thinhorn (*O.dalli*).

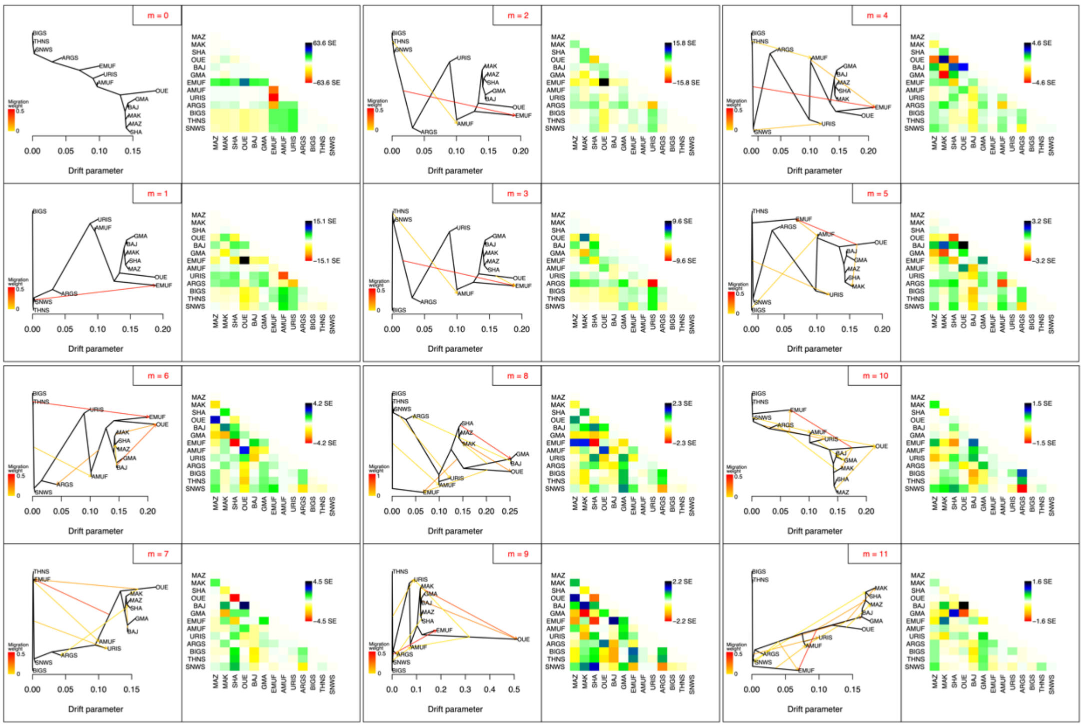

Supplementary Fig. 13 TreeMix inference among *Ovis* species with migration number(m) from 1 to 11, 0 means no migration. Here, BAJ is Baidarak sheep (*O.aries*), GMA is Tibetan sheep (*O.aries*), SHA is Shal sheep (*O.aries*), MAZ is Mazekh sheep (*O.aries*), MAK is Makui sheep (*O.aries*), OUE is Ouessant sheep (*O.aries*), EMUF is European mouflon (*O.musimon*), AMUF is Asiatic mouflon (*O.orientalis*) , URIS is urial (*O.vignei*), ARGS is argali (*O.ammon*), SNWS is snow sheep (*O.nivicola*), BIGS is bighorn (*O.canadensis*), and THNS is thinhorn (*O.dalli*).

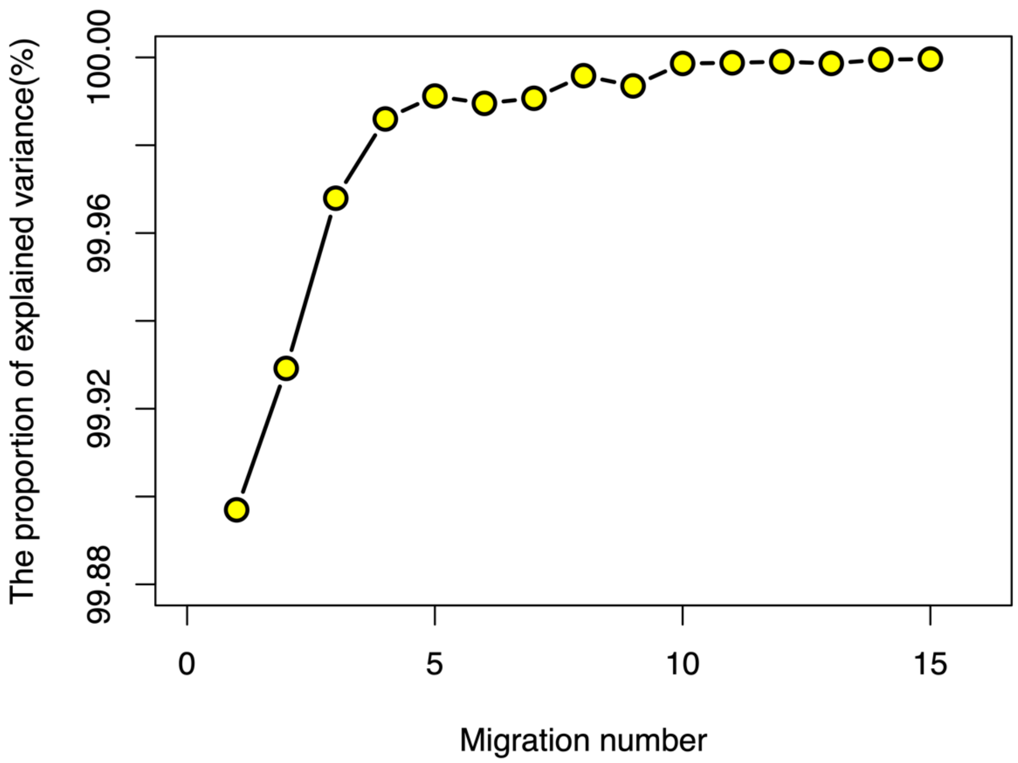

Supplementary Fig. 14 Proportion of explained variance under fifteen model (Migration number from 1 ~ 15). Explained variance remains stable when m=10.

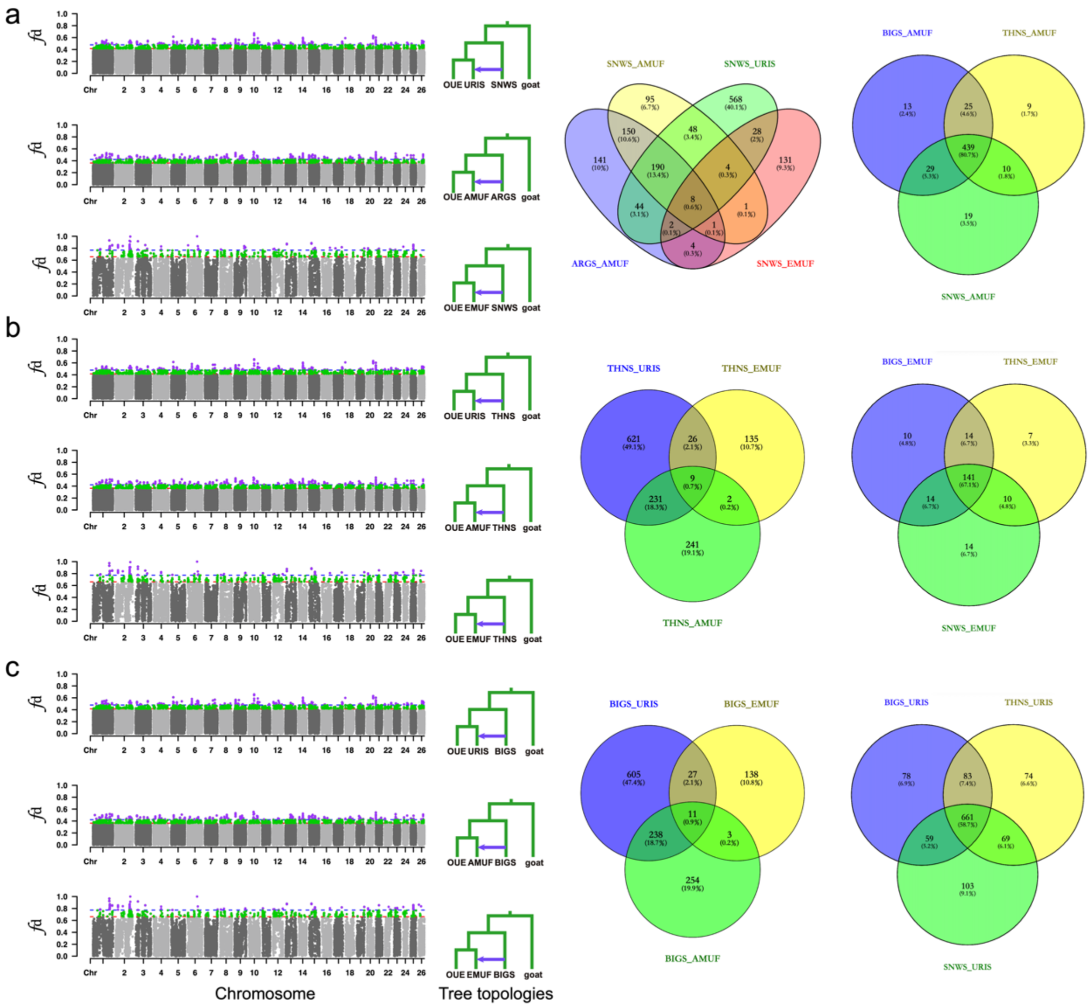

Supplementary Fig. 15 Introgressions into European mouflon genome, Asiatic mouflon, urial from remote lineage of wild sheep (snow sheep or argali, bighorn sheep and thinhorn sheep. Modified *f*-statistic (f_d) for 100-kb windows with 20-kb steps is plotted along the chromosomes. Each dot represents a 100-kb window. Green and blue dots above red horizontal line correspond to FDR 5% and FDR 1% significance level threshold. Number of segments and overlapped genes for three pairs in each group **(a)** [*D*(OUE, test; snow sheep, goat) and *D*(OUE, AMUF; ARGS, goat), **(b)** *D*(OUE, test; thinhorn sheep, goat), **(c)** *D*(OUE, test; bighorn sheep, goat)] were counted and visualized in Manhattan plot and Venn diagram. Source data was presented in Supplementary Data 4.

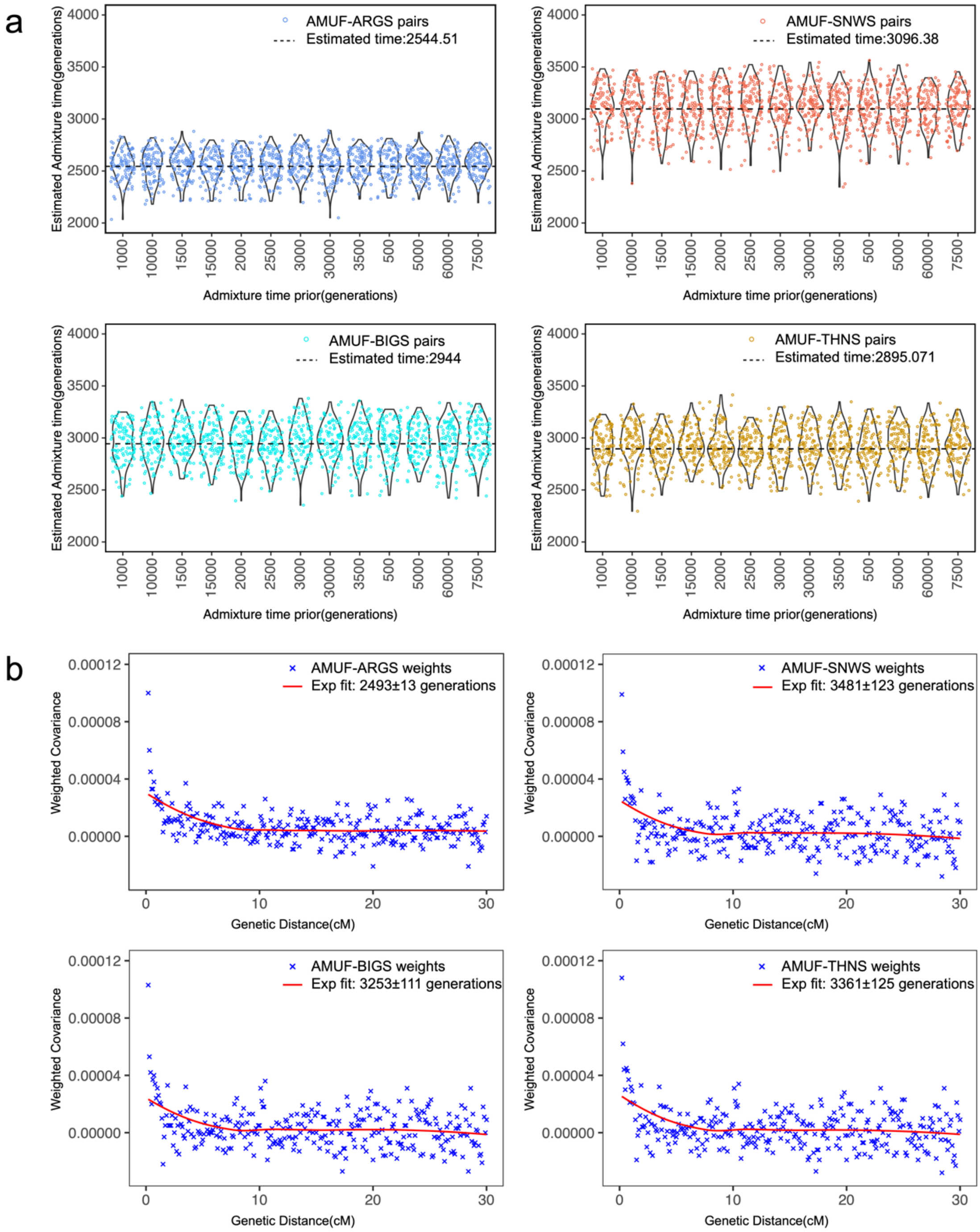

Supplementary Fig. 16 Introgression time inferred by Ancestry_HMM program and DATES software. **(a)** History of introgression estimated of argali (ARGS), snow sheep (SNWS), bighorn sheep (BIGS) and thinhorn sheep (THNS) by Ancestry_hmm program. Prior of admixture time was set from 500~60,000 generations, and 100 bootstrap replicates using a block size of 5000 SNPs was marked in dots of each group.

Dashed line is average time. **(b)** History of introgression estimated by DATES. Weighted LD curves for Asiatic mouflon (AMUF) using argali, snow sheep, bighorn sheep and thinhorn sheep as reference, respectively. Genetic distances are discretized into bins at 0.05 cM resolution. Data for each curve are plotted and fitted starting from the corresponding DATES-computed LD correlation thresholds.

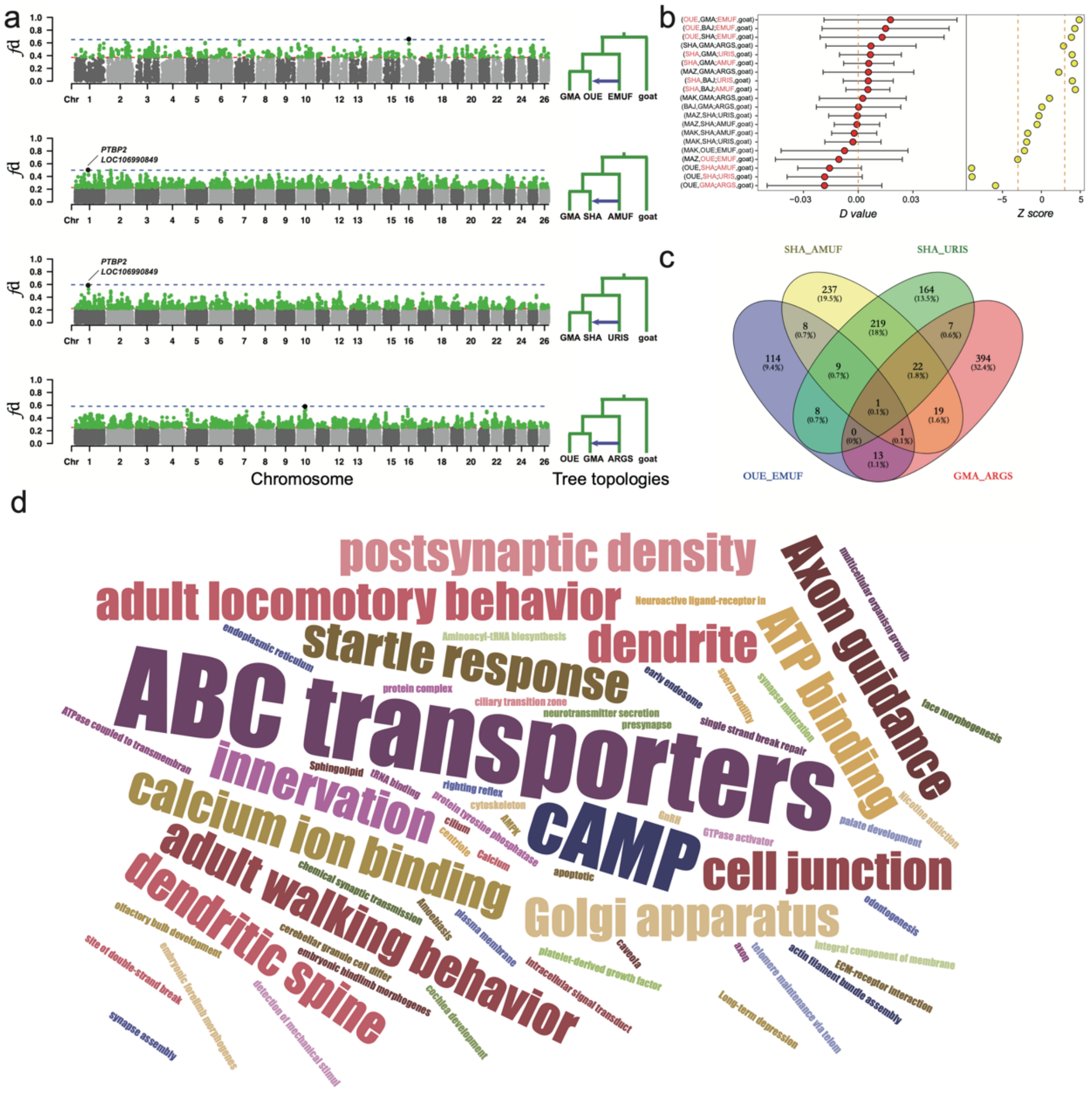

Supplementary Fig. 17 Local introgression from wild sheep to domestic sheep. **(a)** Regions of introgression identified in the domestic sheep. A modified *f*-statistic (f_d) for 100-kb windows with 20-kb steps is plotted along the chromosomes. Each dot represents a 100-kb window. Green and red dots above red horizontal line correspond to FDR 5% and FDR 1% significance level threshold, respectively. **(b)** *D* statistics about pairs between European mouflon (EMUF) and Ouessant sheep (OUE), Asiatic mouflon (AMUF) and Shal sheep (SHA), urial (URIS) and Shal sheep (SHA), argali (ARGS) and Tibetan sheep (GMA). Double dashed line marked as the range of threshold from -3 to 3. **(c)** Number of overlapped genes of four pairs were plotted in

Venn diagram. **(d)** Word cloud for GO terms and KEGG pathway annotated for all the introgressed genes in the four pairs. Source data for **(c, d)** were presented in Supplementary Data 4.

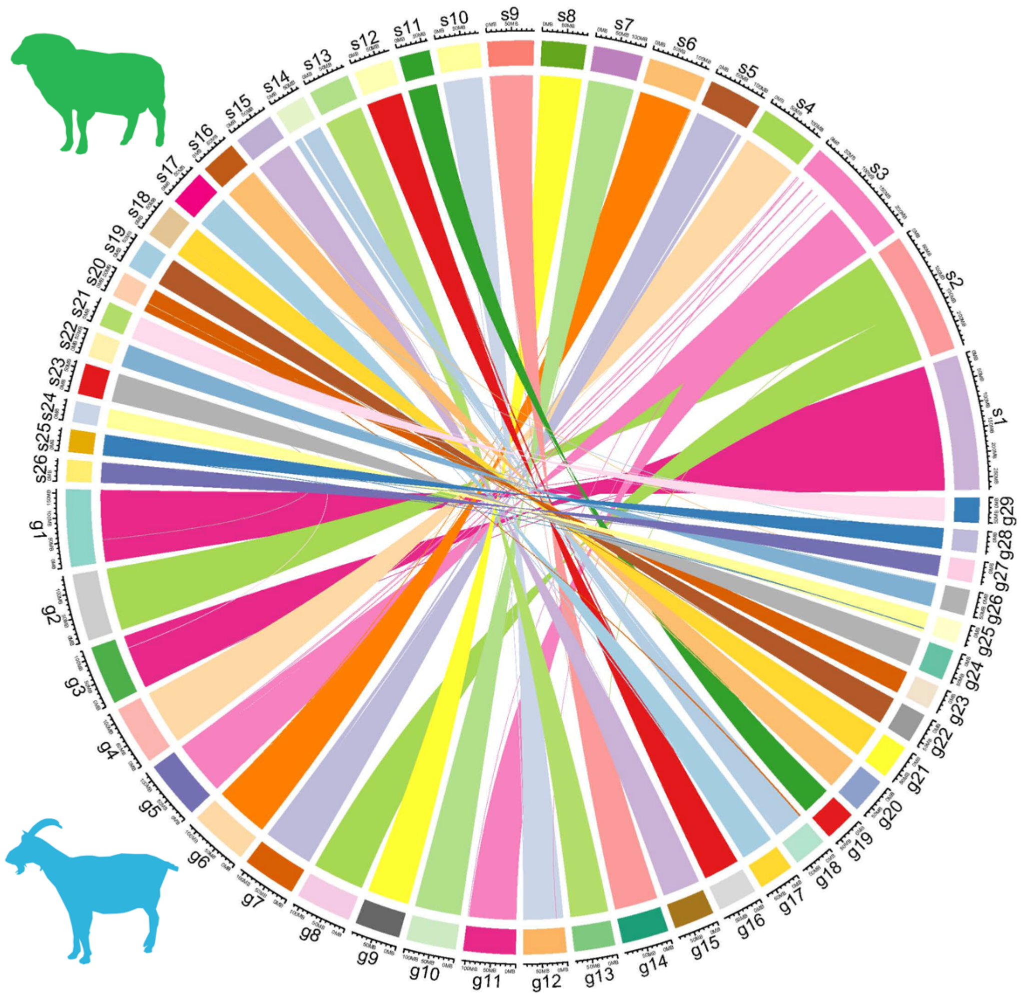

Supplementary Fig. 18 Circos plot of comparison between sheep genome and goat genome by blasting sheep genome to goat genome, only one-to-one orthologs is considered. s1-26 means autosomes of sheep genome, and g1-29 means autosomes of goat genome.

Supplementary Tables

Supplementary Table 1 SNPs Count of *Ovis* genus by chromosome after hard filtering.

Chr	*O.aries*	*O.orientalis*	*O.musimon*	*O.ammon*	*O.vignei*	*O.canadensis*	*O.dalli*	*O.nivicola*
1	3,274,401	5,741,605	1,398,957	2,657,214	3,216,056	2,410,258	2,420,437	2,379,483
2	2,865,550	5,181,137	1,230,826	2,374,565	2,870,863	2,162,325	2,168,774	2,134,818
3	2,569,797	4,579,529	1,091,895	2,132,816	2,556,506	1,951,355	1,967,173	1,948,386
4	1,442,751	2,525,737	653,478	1,194,024	1,415,760	1,068,025	1,077,688	1,059,861
5	1,240,940	2,192,755	589,594	1,027,880	1,245,724	925,041	936,897	921,638
6	1,488,167	2,594,807	639,630	1,202,748	1,470,634	1,103,825	1,095,562	1,082,741
7	1,172,735	2,034,197	510,183	954,963	1,143,228	871,600	876,486	860,549
8	1,088,765	1,886,333	485,599	870,866	1,073,167	799,943	802,625	788,371
9	1,172,102	2,059,245	522,313	939,053	1,177,504	858,374	864,362	852,249
10	1,087,446	1,972,682	574,832	939,575	1,089,415	832,295	835,810	834,658
11	678,727	1,233,469	283,496	602,880	690,234	553,273	556,748	547,611
12	946,712	1,658,820	368,477	783,235	914,203	705,747	716,075	697,077
13	931,369	1,673,251	406,557	764,610	947,177	729,693	731,821	723,081
14	728,738	1,265,887	322,526	624,916	730,368	570,845	578,042	570,809
15	1,007,806	1,754,853	429,701	823,800	957,767	750,337	753,502	742,247
16	926,312	1,600,754	410,859	729,073	923,801	674,668	672,823	661,843
17	890,938	1,528,175	399,181	718,377	884,079	649,530	652,753	642,601
18	834,214	1,465,847	392,862	682,762	834,346	631,547	639,901	623,090
19	688,598	1,231,462	308,434	585,706	702,789	532,153	536,686	524,352
20	653,154	1,117,793	293,381	564,935	639,072	512,071	514,893	506,684
21	645,187	1,128,188	302,089	549,906	660,815	493,689	501,206	491,534
22	634,267	1,100,697	249,777	527,985	624,980	474,175	475,060	466,507
23	796,572	1,348,067	311,916	641,248	752,567	571,899	570,287	558,326
24	492,391	874,622	191,586	413,477	479,992	387,580	396,271	385,012
25	615,717	1,036,906	275,231	489,546	599,041	433,425	436,160	436,817
26	574,145	989,627	238,131	463,829	545,372	422,513	421,313	419,270
A	29,447,501	51,776,445	12,881,511	24,259,989	29,145,460	22,076,186	22,199,355	21,859,615
X	941,296	1,842,240	478,513	900,776	980,443	992,667	985,823	985,506
Mt	36	147	10	106	77	191	196	174
Total	30,388,833	53,618,832	13,360,034	25,160,871	30,125,980	23,069,044	23,185,374	22,845,295

Note:A meas Autosome; X is X chromosome; Mt is Mitochondria.

Supplementary Table 2 Counts of SNPs interactions between species of *Ovis* genus.

**Species**	**The number of SNPs**							
	O.aries	***O.dalli***	***O.canadensis***	***O.nivicola***	***O.orientalis***	***O.vignei***	***O.ammon***	***O.musimon***
O.aries	30,388,833	29,858	29,726	40,589	3,858,323	303,272	168,880	661,840
O.dalli	29,858	23,185,374	1,225,734	173,292	91,402	24,577	47,993	13,535
O.canadensis	29,726	1,225,734	23,069,044	126,900	91,057	24,189	45,763	13,043
O.nivicola	40,589	173,292	126,900	22,845,295	126,806	32,753	87,065	14,431
O.orientalis	3,858,323	91,402	91,057	126,806	53,618,832	4,884,637	1,150,795	217,271
O.vignei	303,272	24,577	24,189	32,753	4,884,637	30,125,980	256,253	73,458
O.ammon	168,880	47,993	45,763	87,065	1,150,795	256,253	25,160,871	45,748
O.musimon	661,840	13,535	13,043	14,431	217,271	73,458	45,748	13,360,034

Note: The total number of SNPs for each species is on the diagonal.

Supplementary Table 3 Counts of INDELs interactions between species of *Ovis* genus.

Species	The number of INDELs							
	O.aries	***O.dalli***	***O.canadensis***	***O.nivicola***	***O.orientalis***	***O.vignei***	***O.ammon***	***O.musimon***
O.aries	4,289,502	1,638,840	1,624,414	1,581,300	3,142,660	2,562,721	1,917,507	1,895,598
O.dalli	1,638,840	4,212,898	3,233,026	2,950,514	2,024,162	1,863,403	2,025,688	1,514,724
O.canadensis	1,624,414	3,233,026	4,166,285	2,934,275	2,007,350	1,848,832	2,011,732	1,507,376
O.nivicola	1,581,300	2,950,514	2,934,275	4,116,471	1,959,214	1,802,901	1,968,258	1,453,955
O.orientalis	3,142,660	2,024,162	2,007,350	1,959,214	7,057,179	3,760,681	2,450,416	2,046,461
O.vignei	2,562,721	1,863,403	1,848,832	1,802,901	3,760,681	4,669,100	2,225,576	1,890,499
O.ammon	1,917,507	2,025,688	2,011,732	1,968,258	2,450,416	2,225,576	4,445,341	1,691,516
O.musimon	1,895,598	1,514,724	1,507,376	1,453,955	2,046,461	1,890,499	1,691,516	3,087,652

Note: The total number of INDELs for each species is on the diagonal.

Supplementary Table 4 The structural variations (SVs) interactions between species of *Ovis* genus.

Species	The number of SVs							
	O.aries	***O.dalli***	***O.canadensis***	***O.nivicola***	***O.orientalis***	***O.vignei***	***O.ammon***	***O.musimon***
O.aries	123,594	48,237	47,680	47,058	91,186	61,342	54,663	48,759
O.dalli	48,237	77,304	58,354	55,067	52,510	40,545	43,419	30,796
O.canadensis	47,680	58,354	75,480	54,582	51,813	40,317	42,947	30,541
O.nivicola	47,058	55,067	54,582	75,375	51,126	40,301	42,259	29,884
O.orientalis	91,186	52,510	51,813	51,126	161,892	69,153	60,396	48,241
O.vignei	61,342	40,545	40,317	40,301	69,153	81,003	45,967	37,928
O.ammon	54,663	43,419	42,947	42,259	60,396	45,967	84,587	34,065
O.musimon	48,759	30,796	30,541	29,884	48,241	37,928	34,065	55,950

Note: The total number of SVs for each species is on the diagonal.

Supplementary Table 5 Annotation of structural variations (SVs) in eight species of *Ovis* genus.

Species	*O.aries*	*O.dalli*	*O.canadensis*	*O.nivicola*	*O.orientalis*	*O.vignei*	*O.ammon*	*O.musimon*
**Sample size**	18	6	6	8	16	7	8	3
**Total**	123,594	77,304	75,480	75,375	161,892	81,003	84,587	55,950
**Exonic**	2,195	1,585	1,520	1,222	3,033	1,259	1,666	1,204
**Percentage(%) of exonic SVs**	1.78%	2.05%	2.01%	1.62%	1.87%	1.55%	1.97%	2.15%
**Intergenic**	81,333	49,893	48,655	49,401	106,941	52,917	55,042	36,054
**Percentage(%) of intergenic SVs**	65.81%	64.54%	64.46%	65.54%	66.06%	65.33%	65.07%	64.44%
**Intronic**	36,569	23,246	22,831	22,621	47,156	24,549	25,115	16,855
**Percentage(%) of intronic SVs**	29.59%	30.07%	30.25%	30.01%	29.13%	30.31%	29.69%	30.13%
**Splicing**	424	258	249	219	684	236	293	199
**Percentage(%) of Splicing**	0.34%	0.33%	0.33%	0.29%	0.42%	0.29%	0.35%	0.36%
**Upstream**	1,462	1,143	1,074	901	1,973	938	1,156	816
**upstream/downstream**	44	43	38	33	54	38	36	31
**Downstream**	1,217	843	834	762	1,629	812	989	541
**Unknowns**	350	293	279	216	422	254	290	250

Supplementary Table 6 Annotation of SNPs in eight species of *Ovis* genus.

Category	Species							
	O.aries	***O.orientalis***	***O.ammo n***	***O.cana densis***	***O.musim on***	***O.nivicol a***	***O.dalli***	***O.vign ei***
**Sample size**	18	16	8	6	3	8	6	7
**Total number of SNPs**	30,388,833	53,618,832	25,160,8 71	23,069, 044	13,360,0 34	22,845,2 95	23,185, 374	30,125, 980
**Intergenic (%)**	67.56%	67.21%	66.92%	66.22%	67.85%	66.33%	66.22%	67.46%
**Intronic (%)**	30.54%	30.90%	31.04%	31.64%	30.10%	31.63%	31.63%	30.72%
**Exonic (%)**	0.633%	0.624%	0.671%	0.717%	0.678%	0.676%	0.720%	0.590%
**Synonymous (%)**	0.355%	0.360%	0.374%	0.399%	0.358%	0.381%	0.399%	0.341%
**Non-synonymous (%)**	0.274%	0.260%	0.293%	0.314%	0.315%	0.292%	0.316%	0.245%
**Non-synonymous/Synonymous**	0.772	0.723	0.785	0.786	0.880	0.766	0.791	0.719
**Splicing (SNP number)**	969	1,345	962	925	719	767	926	871
**Upstream (%)**	0.601%	0.603%	0.649%	0.675%	0.652%	0.627%	0.677%	0.578%
**Downstream (%)**	0.565%	0.572%	0.612%	0.640%	0.592%	0.633%	0.642%	0.565%
**Upstream to downstream (SNP number)**	2,400	4,336	2,276	2,312	1,120	2,089	2,325	2,241
**Ts/Tv**	2487.000	2,43	2.358	2.369	2.255	2.369	2.352	2.391

Note: Ts, transition; Tv, transversion.

Supplementary Table 7 Matrix of pairwise F_{ST} over the eight *Ovis* species.

Species	Pairwise F_{ST}							
	O.aries	***O.orientalis***	***O.musimon***	***O.vignei***	***O.ammon***	***O.nivicola***	***O.dalli***	***O.canadensis***
O.aries	0	0.0944	0.1219	0.1155	0.2177	0.2461	0.2260	0.2261
O.orientalis	0.0944	0	0.1191	0.0470	0.1511	0.1737	0.1540	0.1542
O.musimon	0.1219	0.1191	0	0.2016	0.3553	0.3997	0.3841	0.3867
O.vignei	0.1155	0.0470	0.2016	0	0.2652	0.3153	0.2819	0.2824
O.ammon	0.2177	0.1511	0.3553	0.2652	0	0.2816	0.2689	0.2703
O.nivicola	0.2461	0.1737	0.3997	0.3153	0.2816	0	0.1180	0.1255
O.dalli	0.2260	0.1540	0.3841	0.2819	0.2689	0.1180	0	0.0958
O.canadensis	0.2261	0.1542	0.3867	0.2824	0.2703	0.1255	0.0958	0

Supplementary References

1. Langmesser, S. *et al.* cGMP-dependent protein kinase type I is implicated in the regulation of the timing and quality of sleep and wakefulness. *PloS one* **4**, e4238-e4238 (2009).

2. Gamble, K.L. & Ciarleglio, C.M. Ryanodine receptors are regulated by the circadian clock and implicated in gating photic entrainment. *J. Neurosci.* **29**, 11717-11719 (2009).

3. Morató, X. *et al.* Metabotropic glutamate type 5 receptor requires contactin-associated protein 1 to control memory formation. *Hum. Mol. Genet.* **27**, 3528-3541 (2018).

4. Polimanti, R. *et al.* A genome-wide gene-by-trauma interaction study of alcohol misuse in two independent cohorts identifies *PRKG1* as a risk locus. *Mol. Psychiatry* **23**, 154-160 (2018).

5. Cabana-Domínguez, J. *et al.* Association of the *PLCB1* gene with drug dependence. *Sci. Rep.* **7**, 10110 (2017).
