# Supplementary material for: Whole-genome sequence analysis unveils different origins of European and Asiatic mouflon and domestication-related genes in sheep
Source: Commun Biol. 2021 Nov 18;4:1307. doi: 10.1038/s42003-021-02817-4 (PMC8602413; doi:10.1038/s42003-021-02817-4)
Supplement: Supplementary file 2 — Description of Additional Supplementary Files [file 42003_2021_2817_MOESM2_ESM.pdf]

Description of Additional Supplementary Files

File name: Supplementary Data 1
Description: Sample information and data quality for Ovis species.

File name: Supplementary Data 2
Description: Number of total,unique and common variants (SNPs, INDELs, SVs, CNVs), summary statistics and annotation of SVs among the eight Ovis species.

File name: Supplementary Data 3
Description: Summary information for SNPs identified in the present study with public database and the Ovine BeadChip data, and statistics for experimental validation of SNPs and CNVs.

File name: Supplementary Data 4
Description: Introgression detection by D statistics based on populations and individuals, and Source data for introgressive tracts and genes annotation for pairs of Asiatic mouflon and argali/snow sheep.

File name: Supplementary Data 5
Description: Source data for selection signatures and functions of sixty-two domestication associated genes identified in this study.

File name: Supplementary Data 6
Description: Statistics of ILSs Possibility for pairs of introgression candidates.

File name: Supplementary Data 7
Description: Source data for Figs. 1-3.
