# Supplementary material for: Whole-genome sequence analysis unveils different origins of European and Asiatic mouflon and domestication-related genes in sheep
Source: Commun Biol. 2021 Nov 18;4:1307. doi: 10.1038/s42003-021-02817-4 (PMC8602413; doi:10.1038/s42003-021-02817-4)
Supplement: Supplementary file 10 — Reporting Summary [file 42003_2021_2817_MOESM10_ESM.pdf]

nature portfolio

Corresponding author(s): Meng-Hua Li, Rasmus Nielsen

Last updated by author(s): Sep 22, 2021

Reporting Summary

Nature Portfolio wishes to improve the reproducibility of the work that we publish. This form provides structure for consistency and transparency in reporting. For further information on Nature Portfolio policies, see our Editorial Policies and the Editorial Policy Checklist.

Statistics

For all statistical analyses, confirm that the following items are present in the figure legend, table legend, main text, or Methods section.

n/a	Confirmed	
☐	☒	The exact sample size (*n*) for each experimental group/condition, given as a discrete number and unit of measurement
☐	☒	A statement on whether measurements were taken from distinct samples or whether the same sample was measured repeatedly
☐	☒	The statistical test(s) used AND whether they are one- or two-sided<br>*Only common tests should be described solely by name; describe more complex techniques in the Methods section.*
☒	☐	A description of all covariates tested
☐	☒	A description of any assumptions or corrections, such as tests of normality and adjustment for multiple comparisons
☐	☒	A full description of the statistical parameters including central tendency (e.g. means) or other basic estimates (e.g. regression coefficient) AND variation (e.g. standard deviation) or associated estimates of uncertainty (e.g. confidence intervals)
☐	☒	For null hypothesis testing, the test statistic (e.g. *F*, *t*, *r*) with confidence intervals, effect sizes, degrees of freedom and *P* value noted<br>*Give P values as exact values whenever suitable.*
☒	☐	For Bayesian analysis, information on the choice of priors and Markov chain Monte Carlo settings
☐	☒	For hierarchical and complex designs, identification of the appropriate level for tests and full reporting of outcomes
☒	☐	Estimates of effect sizes (e.g. Cohen's *d*, Pearson's *r*), indicating how they were calculated

Our web collection on statistics for biologists contains articles on many of the points above.

Software and code

Policy information about availability of computer code

Data collection	BWA v0.7.17, Picard v.2.25.4,GATK-v4.0.4.0,ANNOVAR v.2013-06-21,Shapeit v4.1.3, novoBreak v.1.1.3, manta v.1.6.0, GRIDSS v2.6.2,LUMPY v.0.2.13, SpeedSeq v.0.1.2, CNVnator v.0.3.3,SVTyper v.0.1.4,SURVIVOR v.1.0.6,Primer Premier 5, Seqman Sequence Analysis Software Version 6,pairwise sequentially Markovian coalescent (PSMC), SMC++ program, Vcftools v0.1.13, Corrplot package in R version 3.6.1,PopLDdecay v3.30, Smartpca program in EIGENSOFT v7.2.1, Ohana program, RAxML v8.2.3, ANGSD v0.934, Ape package in R version 3.6.1, Densitree v2.0.1, MEGA7,coalescent hidden Markov model (CoalHMM), TreeMix v1.13, LAST v984, Circlize package in R version 3.6.1, SAMtools v.1.3.1, Bcftools v1.5.26, AdmixTools v4480, admixturegraph package in R version 3.6.1, DATES program, Ancestry_hmm program, DAVID v6.8.
Data analysis	This study used publicly available software which is referenced accordingly in the manuscript. Some in-house scripts used for plotting. No customised software was used.

For manuscripts utilizing custom algorithms or software that are central to the research but not yet described in published literature, software must be made available to editors and reviewers. We strongly encourage code deposition in a community repository (e.g. GitHub). See the Nature Portfolio guidelines for submitting code & software for further information.

Data

Policy information about availability of data

All manuscripts must include a data availability statement. This statement should provide the following information, where applicable:

- Accession codes, unique identifiers, or web links for publicly available datasets
- A description of any restrictions on data availability
- For clinical datasets or third party data, please ensure that the statement adheres to our policy

Raw sequencing data that support the findings of this study can be found in the NCBI database under the BioProject accession PRJNA764308, and 38 downloaded

data are under the BioProject accession PRJNA624020 and PRJNA645671. Source data for Supplementary Figs.2, 3, 15 are presented in the Supplementary Data. Additional data such as raw image files and in-house scripts that support this study are available from the first authors upon request.

Field-specific reporting

Please select the one below that is the best fit for your research. If you are not sure, read the appropriate sections before making your selection.

[x] Life sciences [ ] Behavioural & social sciences [ ] Ecological, evolutionary & environmental sciences

For a reference copy of the document with all sections, see nature.com/documents/nr-reporting-summary-flat.pdf

Life sciences study design

All studies must disclose on these points even when the disclosure is negative.

Sample size	No sample-size calculation was performed, but the sample size chosen was comparable to the previous literatures similarly reporting genomic resources for wild species. For instance, Wu et al, 2018 used 11 wisent, 22 gayal and 8 bali, which were the wild relatives of cattle to determine the phylogeny and evolutionary history of the Bos genus. Hu et al. 2018 used 4 argali, 1 European mouflon to detect the genomic traces left by centuries of natural and artificial selection and adaptive introgression from argali in the genomes of Tibetan sheep. Therefore, we think in our study, the sample size of 18 domestic sheep and 54 wild Ovis species [O. musimon (n = 3), O. vignei (n = 7), O. nivicola (n = 8), O. dalli (n = 6) and O. canadensis (n = 6), O. orientalis (n = 16),O. ammon(n = 8)] with high-depth(20-30X) is sufficient to detect demographic history and introgression among Ovis species, and artificial selection between domestic sheep and their wild ancestor (Asiatic mouflon) .
Data exclusions	No data were excluded from the analyses
Replication	All experiments (i.e., PCR add qPCR) were repeated three times with similar results.
Randomization	The random sampling method was used to validate SNPs and CNVs. When did the haploidization of the diploid genomes, we choose bases by randomly selecting, but also verified by choose high quality bases and got the similar results.
Blinding	Blinding was not used because all analyses in the main text were performed on the premise that the samples are known.

Reporting for specific materials, systems and methods

We require information from authors about some types of materials, experimental systems and methods used in many studies. Here, indicate whether each material, system or method listed is relevant to your study. If you are not sure if a list item applies to your research, read the appropriate section before selecting a response.

Materials & experimental systems

n/a	Involved in the study	
[x]	[ ]	Antibodies
[x]	[ ]	Eukaryotic cell lines
[x]	[ ]	Palaeontology and archaeology
[ ]	[x]	Animals and other organisms
[x]	[ ]	Human research participants
[x]	[ ]	Clinical data
[x]	[ ]	Dual use research of concern

Methods

n/a	Involved in the study	
[x]	[ ]	ChIP-seq
[x]	[ ]	Flow cytometry
[x]	[ ]	MRI-based neuroimaging

Animals and other organisms

Policy information about studies involving animals; ARRIVE guidelines recommended for reporting animal research

Laboratory animals	For DNA collection, all laboratory animals (5 samples from 3 breeds generated in this study) are adults. 2 Tibetan sheep, 2 Mazekh sheep and 1 Makui sheep. All is female.
Wild animals	29 out of 54 wild sheep (O.orientalis, O.musimon, O.ammon, O. vignei, O.dalli, O.canadensis, O.nivicola) were generated in this study, including 6 bighorn sheep(O.canadensis), 5 urial sheep(O. vignei), 3 European mouflon(O.musimon), 8 snow sheep(O.nivicola), 1 Asiatic mouflon(O.orientalis) and 6 thinhorn sheep(O.dalli). 20 wild sheep(bighorn, thinhorn and snow sheep) are male, urial sheep are 5 female, 1 Asiatic mouflon and 3 European mouflons are female, Blood or tissue from 29 captive or recently hunted wild sheep (Asiatic mouflon, European mouflon, snow sheep, bighorn, thinhorn and urial) were used for the DNA extraction. After sampling, they were released at the sampling sites.
Field-collected samples	The study did not involve field collected samples.
Ethics oversight	All animal work was conducted according to a permit (No. IOZ13015) approved by the Committee for Animal Experiments of the Institute of Zoology, Chinese Academy of Sciences (CAS), China. For domestic sheep, animal sampling was also approved by local

authorities where the samples were taken.

Note that full information on the approval of the study protocol must also be provided in the manuscript.
